# Pro-C-Type Natriuretic Peptide in Women With Angina Pectoris and No Obstructive Coronary Artery Disease

**DOI:** 10.1016/j.jacadv.2025.101859

**Published:** 2025-06-06

**Authors:** Peter D. Mark, Jakob Schroder, Andreas K. Jensen, Timothy C.R. Prickett, Eva Prescott, Jens P. Goetze

**Affiliations:** a Department of Clinical Biochemistry, Rigshospitalet, University of Copenhagen, Copenhagen, Denmark; b Department of Cardiology, Bispebjerg-Frederiksberg Hospital, University of Copenhagen, Copenhagen, Denmark; c Biostatistics, Department of Public Health, University of Copenhagen, Copenhagen, Denmark; d Department of Medicine, University of Otago, Christchurch, New Zealand; e Department of Biomedical Sciences, Faculty of Health, University of Copenhagen, Copenhagen, Denmark

**Keywords:** CNP, coronary artery disease, C-type natriuretic peptide, natriuretic peptide, NT-proCNP, women

## Abstract

**Background:**

Circulating C-type natriuretic peptides (CNPs) predict adverse outcome in women presenting with ST-elevation myocardial infarction.

**Objectives:**

The purpose of this study was to determine the prognostic impact of a high proCNP concentration in women with angina pectoris but no obstructive coronary artery disease (ANOCA).

**Methods:**

In a prospective cohort of women with ANOCA, we assessed the baseline associations between proCNP concentrations in plasma and clinical data. Moreover, we performed exploratory partial least squares regression (PLS) analyses for correlation patterns of proCNP with 185 cardiovascular plasma markers. We included 1,508 women in baseline/follow-up analyses and 1,598 women in PLS analyses. Follow-up analyses included all-cause death and a composite endpoint of cardiovascular events, where we calculated HR estimates from crude and adjusted (age, creatinine) Cox proportional hazards models.

**Results:**

A high proCNP concentration (223 women) was associated with hypertension (*P* = 0.001), diabetes mellitus (*P* < 0.001), and postmenopausal status (*P* < 0.001) but not age (*P* = 0.13). PLS analyses showed that proCNP concentrations were positively associated with atherosclerotic markers and negatively associated with pro-inflammatory markers. For high proCNP, we found an increased risk of all-cause mortality (HR_crude_: 1.73 [95% CI: 1.10-2.73]; *P* = 0.02 and HR_adjusted_: 1.57 [95% CI: 0.99-2.49]; *P* = 0.06), whereas hazard rates of cardiovascular events were comparable (HR_crude_: 1.08 [95% CI: 0.72-1.62]; *P* = 0.71 and HR_adjusted_: 1.03 [95% CI: 0.68-1.56]; *P* = 0.90).

**Conclusions:**

In women with ANOCA, a high circulating proCNP concentration is associated with a distinct cardiovascular risk profile beyond pro-inflammatory biomarkers and an increased risk of all-cause mortality.

Most patients with angina pectoris but no obstructive coronary artery disease (ANOCA) are women. Despite the absence of macrovascular stenosis, patients with ANOCA have an increased risk of adverse cardiovascular outcomes,^1^, ^2^, ^3^ where coronary microvascular dysfunction (CMD) has been shown to be an independent prognostic factor. Accordingly, studies of plasma biomarkers in patients with ANOCA have focused on identifying molecular targets related to CMD, and several studies have reported up-regulation of pro-inflammatory pathways.^4^, ^5^, ^6^ Nevertheless, the pathogenesis of ANOCA remains largely unresolved, and further understanding of the cardiovascular pathophysiology, including implications of biomarker regulation and effects, is needed to refine strategies for risk stratification and possible intervention.^7^

C-type natriuretic peptide (CNP) is a regulator of cardiovascular homeostasis with protective effects in hypertension and endothelial dysfunction as well as vasodilatory capabilities within the microcirculation.^8^, ^9^, ^10^, ^11^, ^12^ Clinical studies have shown that high plasma concentrations of amino-terminal proCNP (NT-proCNP) and bioactive CNP in circulation are independent predictors of outcome in patients with unstable angina pectoris and chronic heart failure, respectively. Notably, this prognostic value adds to that offered by measurement of atrial and B-type natriuretic peptides (ANP and BNP).^13^, ^14^, ^15^ Recently, we reported on proCNP in plasma from patients presenting with ST-segment elevation myocardial infarction.^16^ In that study, proCNP measurement revealed a marked sex-specific prognostic signal, that is, women but not men with an increased proCNP concentration had an increased risk of all-cause death within 1 year. This female-specific predictive value of proCNP measurement may reflect sex differences in coronary pathophysiology that could potentially be applicable also in women with ANOCA.

In the present study, we measured proCNP in plasma in a large prospective cohort of women with ANOCA. We hypothesized that a high concentration of proCNP is associated with cardiovascular disease and is an independent predictor of all-cause death and adverse cardiovascular events. In addition, we explored patterns of association between proCNP and 185 cardiovascular biomarkers to suggest specific pathophysiological pathways associated with proCNP concentration.

## Methods

### Cohort

The present investigation is a substudy of the iPOWER cohort consisting of women with ANOCA enrolled between March 2012 and December 2017. After screening, women aged up to 80 years with stable or unstable angina referred to coronary angiography with no significant coronary stenosis (>50%) were invited for study participation. Exclusion criteria were absence of chest pain within 6 months of inclusion, suspected nonischemic chest pain, left ventricular ejection fraction <45%, previous verified myocardial infarction or revascularization, moderate/severe heart valve disease, congenital heart disease or cardiomyopathy, severe chronic obstructive pulmonary disease or severe asthma, pregnancy, significant comorbidity or psychiatric disease, language barrier, and travel distance to study site >3 hours. All included women were examined with transthoracic Doppler echocardiography where the coronary flow velocity reserve (CFVR), defined as the ratio between peak and rest flow in the left anterior descending artery, was measured. Peak flow was induced by high-dose dipyridamole (0.84 mg/kg) infusion.^17^^,^^18^ For baseline analyses, we obtained information on medical history, clinical characteristics, echocardiographic parameters, and biochemical measurements as previously described.^17^ To test the association of proCNP with peripheral endothelial function, we included data on reactive hyperemic index evaluated by pulse amplitude tonometry using EndoPAT2000 (Itamar Medical) from an available subset of the cohort.^19^

### Ethics

All participants gave written informed consent. The study was conducted in accordance with the Declaration of Helsinki and was approved by the Danish Regional Committee on Biomedical Research Ethics (Copenhagen) (Ref. H-3-2012-005) and the Danish Data Protection Agency.

### Follow-up

The 2 primary outcomes of interest were all-cause mortality and a composite endpoint (cardiovascular events) defined as the first occurrence of either acute myocardial infarction, heart failure, or stroke based on previous reports.^13^^,^^14^^,^^16^^,^^20^ Noncombined outcomes of acute myocardial infarction, heart failure, and stroke were secondary outcomes. The participants were followed from inclusion until August 18, 2023.

### Biochemical measurement

We measured plasma concentrations of proCNP with a radioimmunoassay based on a processing-independent analysis principle that has been previously reported.^21^^,^^22^ This assay is designed to quantitate the epitope (amino acid 11-27 of the prohormone of proCNP) regardless of potential posttranslational modifications. Relative concentrations of 184 analytes, including BNP and NT-proBNP (given as normalized protein expression values), were determined with Proseek Multiplex INF 96 × 96 panels covering 2 × 92 protein markers using a Proximity Extension Assay technology (Cardiovascular Disease Panels II and III by Olink Proteomics).^23^ See Supplemental Appendix for more details on biochemical measurements.

### Statistics

Based on measured plasma concentrations of proCNP, we divided the cohort into 2 groups of nonhigh and high proCNP concentration, respectively, defined by the cutoff value obtained by the Youden index for maximation of sensitivity and specificity of all-cause mortality based on the findings of this endpoint in our previous study.^16^ Across the 2 groups, we evaluated differences in median values of quantitative variables by Mood’s median test and for categorical variables by Fisher exact *t*-test. Spearman correlation analyses were performed to assess the continuous relation of proCNP concentration and CFVR.

To identify specific molecular targets and pathophysiological pathways associated with proCNP concentration, we explored patterns of association between proCNP and 185 cardiovascular plasma markers (high-sensitivity C-reactive protein [Hs-CRP] and 184 markers of Olink Cardiovascular Panel II and III) by partial least squares regression (PLS).^24^ PLS is a regression model suitable for situations where the matrix of predictors is both high-dimensional and highly collinear. Like principal component analysis, PLS aims to construct a smaller number of new predictors (scores) consisting of linear combinations of the original predictors but under the constraint that they are uncorrelated to each other. These scores can then be associated to the outcome in a linear regression model. Contrary to principal component analysis, PLS specifically aims at constructing the scores so that they are also maximally correlated with the outcome. A PLS analysis including all 185 markers was first performed, and the optimal model with the smallest number of scores was selected according to the leave-one-out cross-validated root-mean-squared error of prediction. While PLS gives low weights to unimportant predictors in its scores, it does not perform variable selection by eliminating predictors. To reduce the 185 target predictors to a smaller number leading to increased interpretability of the scores, we combined PLS with the Boruta algorithm^25^ as a prescreening step. The predictors selected by Boruta were then applied in a new cross-validated PLS model, and a final determination of important predictors was done by bootstrapping the PLS model and selecting those predictors with a nonzero containing 95% CI for their effect sizes.

For follow-up analyses, we depicted the cumulative incidence rates of primary and secondary outcomes in groups of nonhigh and high proCNP concentrations with all-cause mortality as competing risk for cardiovascular events and individual outcomes. We constructed crude and adjusted Cox proportional hazard models to obtain estimates of HR, 95% CIs, and *P* values with cause-specific estimates in the setting of competing risk. In adjusted Cox proportional hazards models, we adjusted for age and plasma creatinine concentration given their association to proCNP-derived peptides in plasma.^26^ Model assumptions of proportional hazards and linearity for proCNP as a continuous variable were visually evaluated by cumulative residual plots and cubic spline plots, respectively. Statistical analyses were performed with R statistical software R version 3.6.1 (R Core Team).^27^

## Results

### Associations of high ProCNP

Of 1,681 women with completed CFVR examination, we included 1,508 women with available plasma, a measured proCNP concentration and available follow-up data. The optimal cutoff value between nonhigh and high proCNP concentrations was determined to be 53.4 pmol/L. In Table 1, the results of baseline clinical characteristics, measures from cardiac and vascular examinations, biochemical analytes, disease prevalence, and incidence of outcomes during follow-up are shown across the 2 groups. A high proCNP concentration was associated with a higher prevalence of hypertension (*P* = 0.001), diabetes (*P* < 0.001), and postmenopausal status (*P* < 0.001), as well as higher plasma concentrations of creatinine (*P* < 0.001) and hemoglobin A1c (*P* < 0.001), and a higher prevalence of increased filling pressure (E/e’ ≥12) (*P* = 0.038). The risk of chance findings should be taken into consideration given the multiple comparisons performed in Table 1. The association of proCNP and peripheral endothelial function was tested in a subset of 306 women (271 with nonhigh and 36 with high proCNP) by using an established cutoff of reactive hyperemic index of <1.67 defining endothelial dysfunction. The prevalence of endothelial dysfunction was 26% vs 38% (*P* = 0.11) in women with nonhigh vs high proCNP, respectively.^19^

**Table 1:** Baseline Characteristics

	All Women With Measured proCNP Concentration			
	N	Nonhigh proCNP (<53.4 pmol/L)	High proCNP (≥53.4 pmol/L)	*P* Value
Number, n (%)	1,508	1,285 (85.2%)	223 (14.8%)	-
Age, y	1,508	63.8 (55.3-69.9)	65.0 (58.6-71.2)	0.13
Height, cm	1,493	166 (162-170)	165 (162-170)	0.99
Weight, kg	1,501	72 (63-83)	72 (65-83)	0.72
BMI, kg/m^2^	1,487	26.1 (23.3-30.1)	26.4 (23.5-29.7)	0.29
Abdominal circumference, cm	1,385	96 (86-105)	98 (88-105)	0.098
Smoking - never, n (%)	1,497	534 (41.8%)	92 (42.0%)	0.55
Smoking - former, n (%)		539 (42.2%)	86 (39.3%)	
Smoking - active, n (%)		205 (16.0%)	41 (18.7%)	
Postmenopausal, n (%)	1,495	1,052 (82.5%)	241 (93.6%)	<0.001
Hormone replacement therapy during menopause, n (%)	1,311	371 (33.6%)	63 (30.4%)	0.42
Active systemic hormone therapy, n (%)	1,495	132 (10.7%)	24 (10.8%)	0.91
Coronary atherosclerosis, n (%)	1,474	451 (37.2%)	107 (43.8.6%)	0.069
Heart rate, beats/min	1,406	69 (63-77)	71 (64-78)	0.16
Systolic blood pressure, mm Hg	1,401	129 (116-140)	131 (119-144)	0.38
Diastolic blood pressure, mm Hg	1,403	66 (60-76)	68 (59-79)	0.37
Echocardiography, median (IQR)				
Left ventricular ejection fraction, %	1,410	58 (53-63)	59 (54-63)	0.82
E/e’ ≥12, n (%)	1,325	123 (11.5%)	31 (17.0%)	0.038
Global longitudinal strain, %	1,329	21.1 (19.3-22.8)	20.8 (18.9-22.4)	0.06
CFVR	1,508	2.35 (2.00-2.76)	2.31 (1.97-2.70)	0.28
CFVR <2.25, n (%)	1,508	542 (42.2%)	102 (45.8%)	0.34
Medical history, n (%)				
Hypertension	1,500	649 (52.8%)	140 (63.6%)	0.001
Diabetes	1,501	119 (9.3%)	63 (26.7%)	<0.001
Peripheral artery disease	1,495	83 (6.5%)	9 (4.1%)	0.22
Cerebrovascular disease	1,496	100 (7.8%)	17 (7.7%)	>0.99
Biochemical analyses, median (IQR)				
Creatinine, μmol/L	1,488	64 (57-73)	68 (60-78)	<0.001
HbA1c, mmol/mol	1,477	38 (35-41)	39 (36-44)	<0.001
Total cholesterol, mmol/L	1,492	5.0 (4.3-5.8)	4.8 (4.1-5.5)	0.083
LDL cholesterol, mmol/L	1,485	2.7 (2.1-3.5)	2.5 (1.8-3.1)	0.048
Triglyceride, mmol/L	1,489	1.1 (0.8-1.5)	1.2 (0.9-1.7)	0.34
TSH, mIU/L	1,320	1.32 (0.84-1.98)	1.19 (0.77-1.89)	0.024
TSH <0.4 mIU/L, n (%)	1,320	72 (6.4%)	17 (8.5%)	0.28
Hs-CRP, mg/L	1,490	1.7 (0.9-3.1)	1.7 (1.0-3.0)	0.65
Hs-CRP >2 mg/L, n (%)	1,490	532 (42.0%)	96 (43.2%)	0.77
NT-proBNP^a^	1,411	3.5 (2.8-4.3)	3.7 (2.8-4.4)	0.26
Follow-up, n (%)				
All-cause death	1,508	83 (6.5%)	24 (10.8%)	0.032
Acute myocardial infarction	1,508	37 (2.9%)	8 (3.6%)	0.53
Heart failure	1,508	50 (3.9%)	9 (4.0%)	0.85
Stroke	1,508	79 (6.1%)	12 (5.3%)	0.76

BMI = body mass index; CFVR = coronary flow velocity reserve; Hs-CRP = high-sensitivity C-reactive protein; LDL = low-density lipoprotein; NT-proBNP = amino-terminal pro-B-type natriuretic peptide; TSH = thyroid-stimulating hormone.

a Values are given as normalized protein expression.

### Partial least squares regression analyses

**Figure 1:**
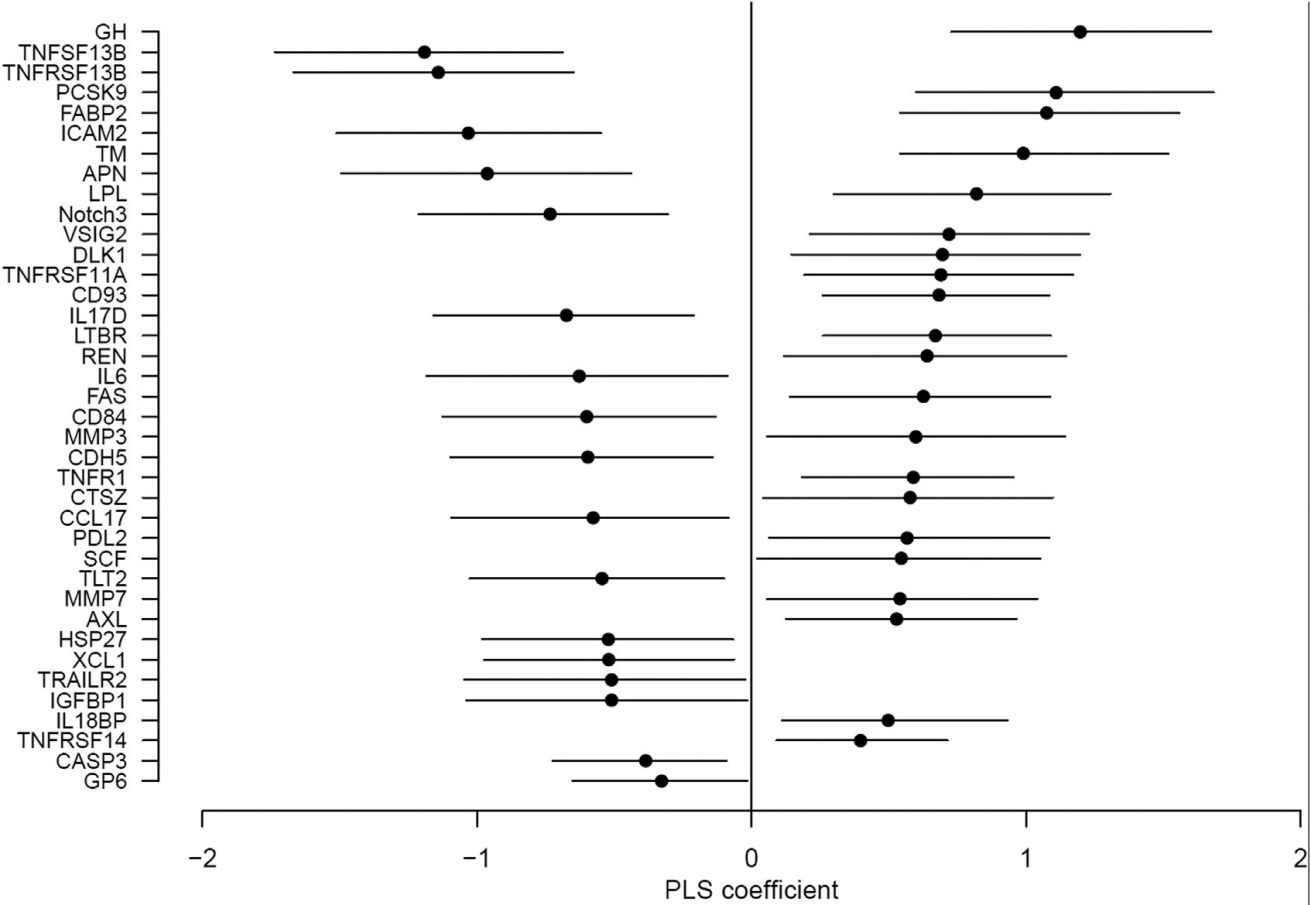
Partial Least Squares Regression Coefficients for 38 Plasma Markers With Association With ProCNP Error bars show bootstrapped 95% CIs of PLS coefficients as indication of the effect size. APN = aminopeptidase N; AXL = tyrosine-protein kinase receptor UFO; CASP3 = caspase 3; CCL17 = CC chemokine ligand 17; CD84 = Cluster of Differentiation 84; CD93 = Cluster of Differentiation 93 antigen; CDH5 = cadherin 5; CTSZ = cathepsin z; DLK1 = protein delta homolog 1; FABP2 = fatty acid-binding protein 2; FAS = Fas receptor; GH = growth hormone; GP6 = glycoprotein VI; HSP27 = heat shock protein 27; ICAM = intracellular adhesion molecule; IGFBP1 = insulin-like growth factor-binding protein 1; IL = interleukin; LPL = lipoprotein lipase; LTBR = lymphotoxin beta receptor; MMP = matrix metalloproteinase; Notch3 = neurogenic locus notch homolog protein 3; PCSK9 = proprotein convertase subtilisin/kexin type 9; REN = renin; PD-L2 = programmed death 1 ligand 2; SCF = stem cell factor; TLT2 = TREM-like transcript 2; TM = thrombomodulin; TNFR1 = tumor necrosis factor receptor 1; TRAILR2 = TRAIL receptor 2; VSIG2 = V-set and immunoglobulin domain containing 2; XCL1 = chemokine ligand 1.

We included 1,598 women with measured proCNP and 185 other cardiovascular biomarkers in PLS analyses. In the full PLS model, the 185 markers could be summarized into 6 uncorrelated scores according to the cross-validation criterion. After applying the variable selection procedure, these were reduced to 113 markers confirmed, 66 were confirmed unimportant, and the status of 6 markers was left undetermined. In the final PLS model, we identified 38 plasma markers with a PLS coefficient different from zero (see Figure 1). The final PLS model showed a modest predictive potential of the observed proCNP concentrations (summary results of PLS analyses are shown in the Supplemental Appendix). The prediction error for the reduced set of variables was, however, comparable to using all 185 markers. The 21 positively associated markers, ordered by strength of association, were growth hormone (GH), proprotein convertase subtilisin/kexin type 9, fatty acid-binding protein 2, thrombomodulin, lipoprotein lipase, V-set and immunoglobulin domain containing 2, protein delta homolog 1, tumor necrosis factor receptor superfamily member 11a and 14 (TNFRSF11A/RANK and TNFRSF14, respectively), Cluster of Differentiation 93 antigen (a C-type lectin transmembrane receptor), lymphotoxin beta receptor, renin, Fas receptor, matrix metalloproteinase (MMP)-3 and -7, tumor necrosis factor receptor 1 (TNFR1), cathepsin z, programmed death 1 ligand 2, stem cell factor, tyrosine-protein kinase receptor UFO (AXL), and interleukin (IL)-18 binding protein. The 17 negatively associated markers were tumor necrosis factor ligand/receptor superfamily member 13b (TNFSF13B/BAFF and TNFRSF13B, respectively), intracellular adhesion molecule 2, aminopeptidase N, neurogenic locus notch homolog protein 3, IL-6 and -17D, Cluster of Differentiation 84, cadherin 5, CC chemokine ligand 17, TREM-like transcript 2, heat shock protein 27, chemokine ligand 1 (or lymphotactin), TRAIL receptor 2, insulin-like growth factor-binding protein 1, caspase 3, and glycoprotein VI.

### Follow-up

**Figure 2:**
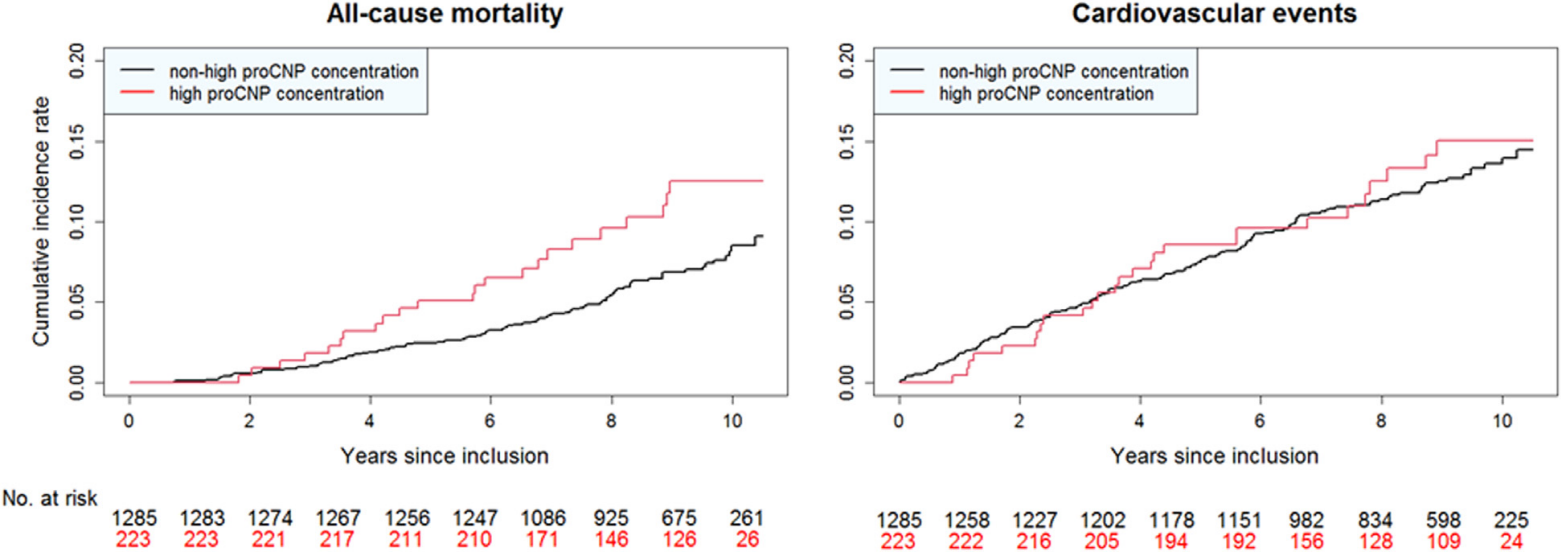
The Cumulative Incidence Rates of the Primary Outcomes

The median duration of follow-up was 9.1 years (7.5-9.9) for all-cause mortality and 8.8 years (6.8-9.9) for cardiovascular events, and 107 deaths of any cause and 175 cardiovascular events occurred (Figure 2, Table 2). A high proCNP concentration was associated with an increased risk of all-cause death (HR_crude_: 1.73 [95% CI: 1.10-2.73]; *P* = 0.02 and HR_adjusted_: 1.57 [95% CI: 0.99-2.49]; *P* = 0.06), whereas the risk of cardiovascular events did not differ between the 2 groups (HR_crude_: 1.08 [95% CI: 0.72-1.62]; *P* = 0.71 and HR_adjusted_: 1.03 [95% CI: 0.68-1.56]; *P* = 0.90). We found no differences in hazard rates of secondary outcomes (Table 2).

**Table 2:** HRs of High Vs NonHigh ProCNP in Crude and Adjusted Cox Proportional Hazards Models

	Crude (1,508 Included in Analyses)			Adjusted^a^ (1,488 Included in Analyses^b^)		
	Events	HR (95% CI)	*P* Value	Events	HR (95% CI)	*P* Value
All-cause death	107	1.73 (1.10-2.73)	0.02	107	1.57 (0.99-2.49)	0.06
Cardiovascular events	175	1.08 (0.72-1.62)	0.71	173	1.03 (0.68-1.56)	0.90
Acute myocardial infarction	45	1.27 (0.58-2.72)	0.55	44	1.27 (0.59-2.78)	0.54
Heart failure	59	1.07 (0.53-2.17)	0.86	57	0.99 (0.48-2.04)	0.99
Stroke	91	0.90 (0.49-1.65)	0.73	91	0.86 (0.47-1.59)	0.63

a Adjusted models are adjusted for age and plasma creatinine concentration (both continuous).

b Due to missing values of creatinine concentrations, fewer subjects are included in the adjusted models.

## Discussion

In this study of >1,500 women with ANOCA, a high concentration of proCNP was associated with a higher baseline prevalence of hypertension, diabetes mellitus, and postmenopausal status as well as higher concentrations of creatinine and increased filling pressure (E/e’ ≥12). Furthermore, a high proCNP concentration was not associated with increased concentrations of NT-proBNP or sign of low-grade inflammation (Hs-CRP >2 mg/L). This combination of risk factors is consistent with our previous study of proCNP measurement in women with ST-elevation myocardial infarction despite the differences in disease severity and age.^16^ In accordance, other reports have also linked high concentrations of circulating proCNP-derived peptides to a pattern of vascular risk factors.^13^^,^^26^^,^^28^^,^^29^ The association between high proCNP concentrations and diabetes in women is notable, as diabetes is associated with an excess risk of >40% of fatal ischemic heart disease in women compared with men.^30^^,^^31^ Our findings thus raise the question whether increases in proCNP among elderly women are part of an adaptive vascular response to cardiovascular risk factors after menopause. Taken together, the baseline associations of the present study show high proCNP concentration in women with ANOCA is associated with a cardiovascular risk profile independent of NT-proBNP and low-grade inflammation (Central Illustration).

**Central Illustration:**
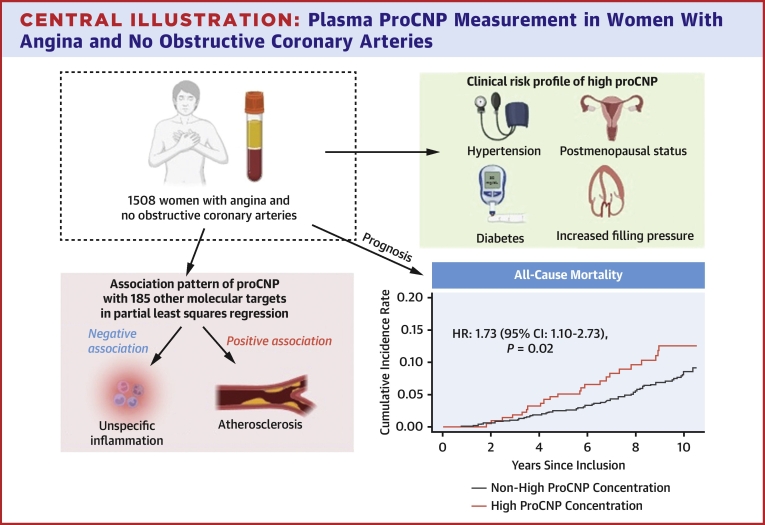
Plasma ProCNP Measurement in Women With Angina and No Obstructive Coronary Arteries

**Figure 3:**
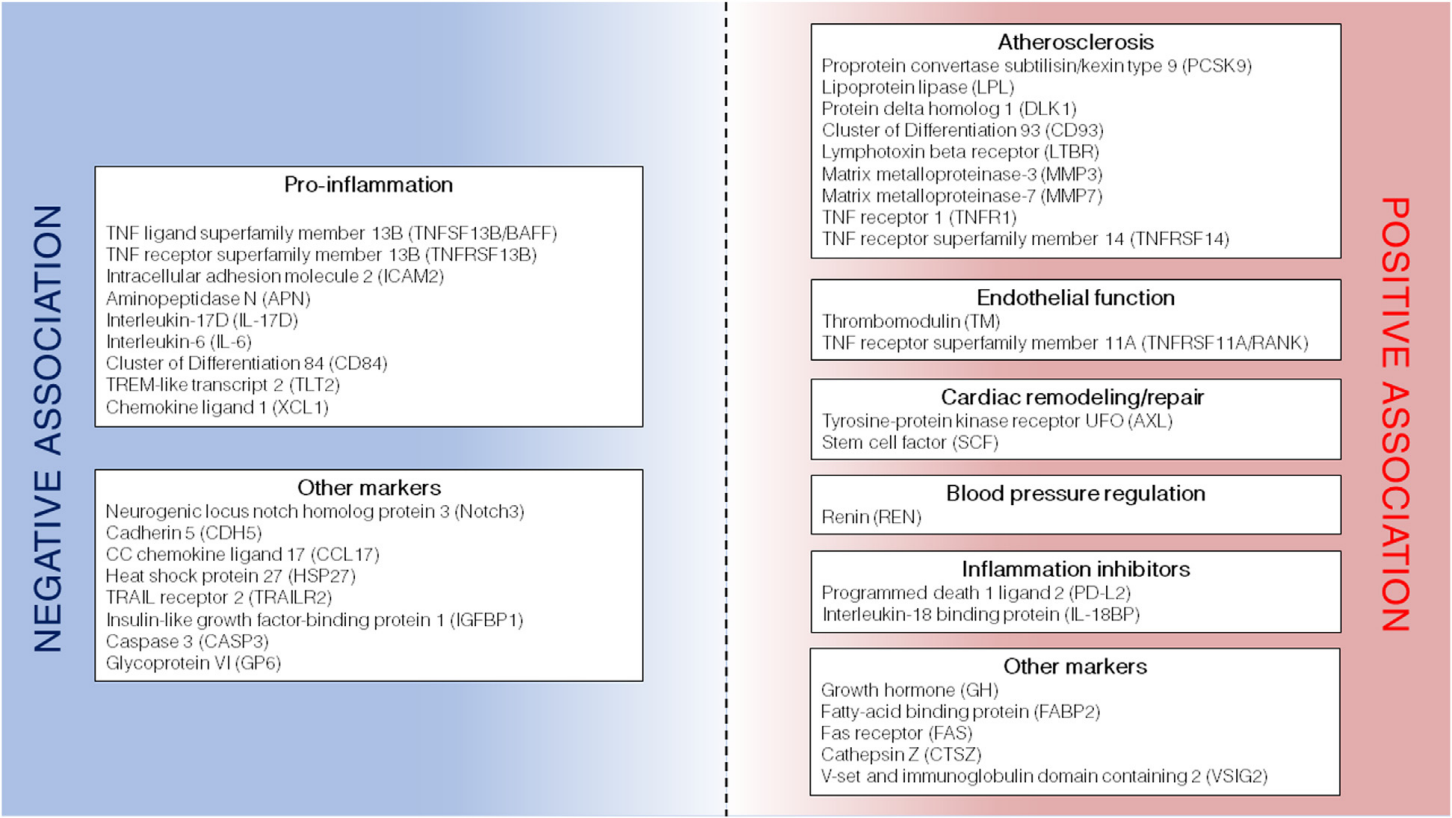
Plasma Markers Associated With ProCNP in Sparse Partial Least Squares Regression Analysis The markers are categorized in functional domains of cardiovascular (patho-) physiology as a schematic overview.

In the exploratory PLS analyses of 185 cardiovascular markers in plasma, we found 38 markers with substantial association to proCNP concentrations. In Figure 3, the markers are crudely divided into groups of (patho-)physiological function. Among the positively associated markers are proteins involved in lipid metabolism and/or pathogenesis of atherosclerosis including proprotein convertase subtilisin/kexin type, lipoprotein lipase, protein delta homolog 1, Cluster of Differentiation 93 antigen, lymphotoxin beta receptor, MMP3, MMP7, TNFR1, and TNFRSF14.^32^, ^33^, ^34^, ^35^, ^36^, ^37^, ^38^, ^39^, ^40^ In addition, proCNP concentrations associate with the endothelial cell marker thrombomodulin (as previously described^16^), the receptor RANK involved in osteoclastic activity and endothelial dysfunction, blood pressure regulating enzyme renin, the receptor AXL and stem cell factor involved cardiac remodeling and repair, respectively, and anti-inflammatory proteins programmed death 1 ligand 2 and IL-18BP.^41^, ^42^, ^43^, ^44^, ^45^ Conversely, the negatively associated markers are dominated by diverse pro-inflammatory proteins including TNFSF13B, TNFRSF13B, intracellular adhesion molecule 2, aminopeptidase N, IL-17D, IL-6, Cluster of Differentiation 84, TREM-like transcript 2, and chemokine ligand 1.^46^, ^47^, ^48^, ^49^, ^50^, ^51^, ^52^, ^53^, ^54^ Interestingly, GH displays the strongest positive association with proCNP. GH plays a number of significant roles in bone growth and endothelial function and instigates protective effects against cardiovascular disease.^55^ Collectively, the pattern of association shows that increasing proCNP concentrations in plasma may reflect aspects of atherogenesis, endothelial function, and blood pressure, which is in accordance with preclinical findings on the regulatory role of CNP in cardiovascular disease.^10^^,^^11^ Furthermore, proCNP displays an inverse relation with a cluster of pro-inflammatory targets and is not associated with markers such as BNP/NT-proBNP and Hs-CRP. Thus, our findings suggest that measurement of CNPs may represent a signal of specific pathophysiological processes in the coronary vasculature distinct from unspecific inflammation. Whether this signal precedes any effect on the traditional natriuretic peptides (ANP and BNP) used in heart failure evaluation remains speculative for now.

The follow-up analyses showed an increased hazard rate of all-cause death of 57% to 73% for women with a high proCNP (*P*_*crude*_ = 0.02 and *P*_*adjusted*_ = 0.06), whereas hazard rates were similar for combined cardiovascular events (*P*_*crude*_ = 0.71 and *P*_*adjusted*_ = 0.90), despite a considerably higher number of events (175 cardiovascular events vs 107 all-cause deaths). This lack of increased risk of cardiovascular events was surprising considering the distinct cardiovascular risk profile identified in this study and the previous reports of circulating CNPs as predictors of adverse cardiovascular events. In particular, we had expected a prognostic value with regard specifically to acute myocardial infarction in view of the many positively associated atherosclerotic markers in the PLS analysis. However, only 45 acute myocardial infarctions occurred and, hence, a longer follow-up time is needed to properly assess the prognostic impact as demonstrated by a previous study of the general population.^13^^,^^14^^,^^20^ A possible explanation of the prognostic findings could be that high proCNP reflects an increased but nonspecific prognostic signal in women with ANOCA not captured by usual cardiovascular events.

Despite the plethora of proposed plasma biomarkers in cardiovascular disease, the clinical value is, for now, largely restricted to the measurement of cardiac troponin and cardiac natriuretic peptides (ANP and BNP). However, the utility of these biomarkers is predominantly for exclusion of acute myocardial damage and heart failure, respectively. For patients with ANOCA, previous studies of plasma biomarkers have examined the relation of specific molecular targets and CMD measured by endothelium-independent vasodilatation by either transthoracic Doppler echocardiography or positron emission tomography.^4^^,^^5^^,^^56^ Collectively, these studies have identified markers of the pro-inflammatory TNF-α–IL-6–CRP pathway to be associated with CMD.^4^^,^^5^ Our results show that proCNP concentration is not associated with endothelium-independent CFVR, low-grade inflammation (Hs-CRP >2 mg/L), or BNP/NT-proBNP and is, in fact, inversely related with IL-6 and a range of other pro-inflammatory markers in PLS analyses. Notably, several preclinical studies have reported an anti-inflammatory role of CNP including down-regulation of interferon-gamma induced gene-expression in human endothelial cells via a cyclic GMP-mediated pathway.^57^, ^58^, ^59^, ^60^ Whether our findings reflect such an anti-inflammatory effect in a clinical context needs further investigation. In summary, the present findings in women with ANOCA suggest that proCNP measurement captures a signal of pathophysiological processes related to atherosclerosis beyond measures of endothelium-independent CMD and markers of general inflammation in plasma. Further clinical studies are needed to thoroughly assess the incremental predictive value of measurement of circulating CNPs as early-stage markers of vascular integrity/cardiac abnormalities with a specific focus on sex-specific analyses.

### Study Limitations

Only women were examined in this study, and whether the findings are mirrored in men with ANOCA could not be evaluated. Regarding PLS analyses, a limiting aspect in addition to its exploratory nature, was that the biomarkers, apart from proCNP and Hs-CRP, were quantified as relative plasma levels (not absolute concentrations). Moreover, many of the measured molecular targets are involved in several physiological processes, and some of the functional domains in Figure 3 are inherently related, for example, atherosclerosis and inflammation. Consequently, the division of correlated markers into separate functional domains is simplified and merely represents a schematic overview of correlation patterns. Hence, these exploratory findings warrant a cautious interpretation.

## Conclusions

High plasma concentrations of proCNP in women with ANOCA are associated with a cardiovascular risk profile including hypertension, diabetes, and postmenopausal status. From PLS analyses of 185 molecular targets, we found a biochemical association pattern of proCNP measurement dominated by atherosclerotic and endothelial markers as well as an inverse relation to a range of pro-inflammatory markers. Notably, this outline of clinical and biochemical risks was beyond measures of endothelium-independent coronary flow reserve as well as plasma concentrations of BNPs and Hs-CRP. In a prognostic perspective, a high proCNP concentration was associated with increased risk of all-cause death but not cardiovascular events.

Perspective **COMPETENCY IN TRANSLATIONAL OUTLOOK:** Measurement of pro-CNP holds prognostic information in women with ANOCA in terms of all-cause mortality.

**TRANSLATIONAL OUTLOOK:** Biomarkers helping the clinician when treating this group of patients are highly warranted to help patients in need—and not harm those not in need.

## Funding support and author disclosures

This work was supported by the Danish Biotek program (a grant from the Danish Health Ministry). The senior author (Dr Goetze) has served as a consultant for Novo Nordisk on biochemical method development. All other authors have reported that they have no relationships relevant to the contents of this paper to disclose.
