# Supplementary material for: Pro-C-Type Natriuretic Peptide in Women With Angina Pectoris and No Obstructive Coronary Artery Disease
Source: JACC Adv. 2025 Jun 6;4(7):101859. doi: 10.1016/j.jacadv.2025.101859 (PMC12177164; doi:10.1016/j.jacadv.2025.101859)

**Supplemental Appendix**

**METHODS**

BIOCHEMICAL MEASUREMENT

Blood samples were obtained at inclusion. Blood was collected in EDTA tubes, centrifuged at 3500 rpm for 10 minutes, and plasma was stored at -80ºC. Prior to measurement of proCNP concentrations in plasma, the samples are treated with trypsin, which releases the epitope from both intact proCNP and the N-terminal fragments. The inter-assay coefficient of variation for proCNP was 4.2% at 40 pmol/L. Concentrations of creatinine, HbA1c, total and low-density cholesterol, triglycerides, thyroid-stimulating hormone (TSH), and high-sensitivity C-reactive protein (Hs-CRP) were obtained through routine measurement in the Departments of Clinical Biochemistry at Bispebjerg Hospital and Rigshospitalet, Copenhagen, Denmark.

**RESULTS**

PARTIAL LEAST SQUARES REGRESSION (PLS) ANALYSES

The relative importance of all 185 markers in the full PLS analysis are listed in **Supplemental Figure 1**. A scatterplot of the predictive potential of the final PLS model is shown in **Supplemental Figure 2**.

**Supplemental Figure 1: Importance of 185 Markers in Full PLS Analysis.**

**
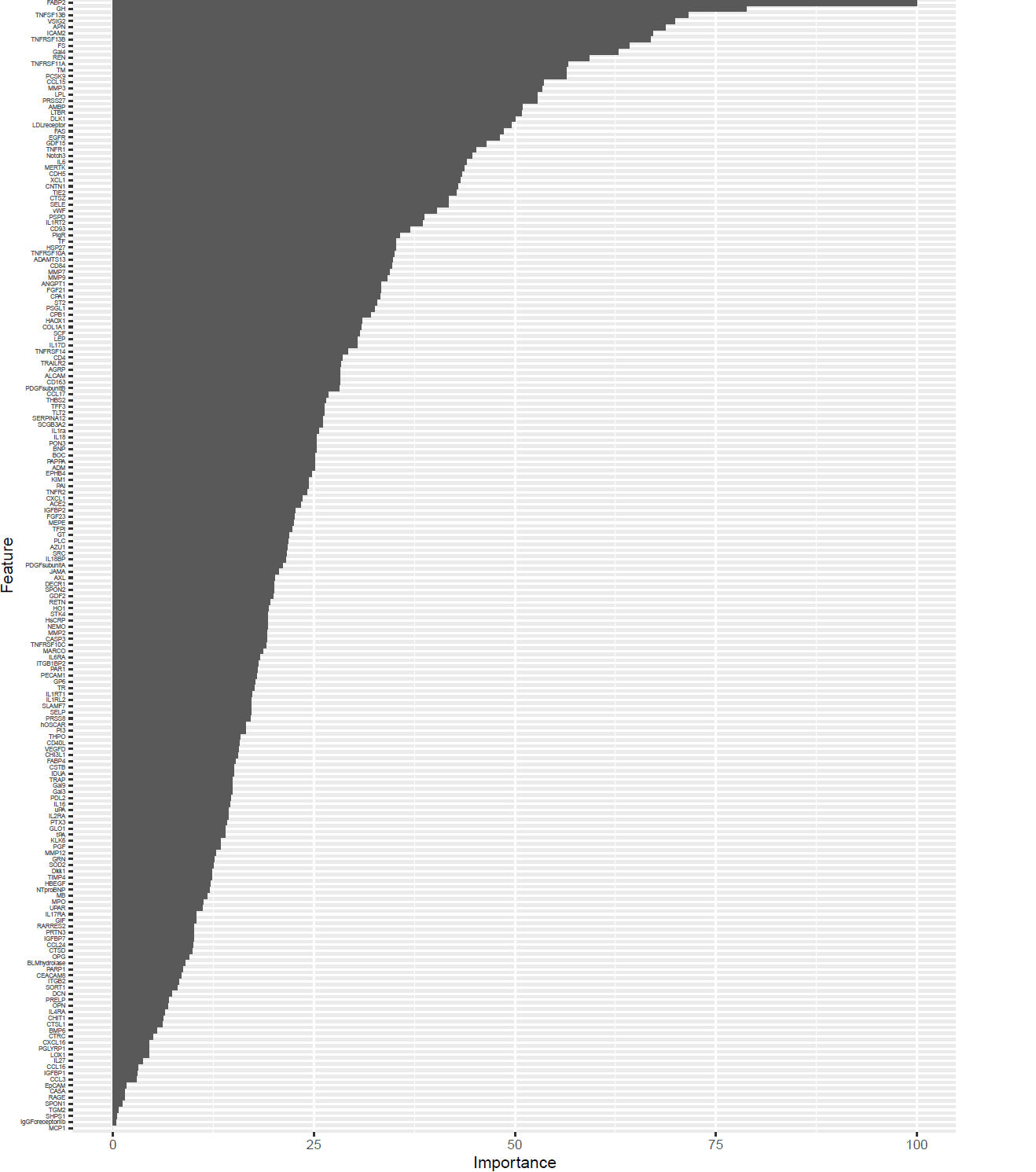
**

The importance is expressed as percent (%) on the x-axis with highest importance of association defined as 100%. The markers are listed in descending order of importance.

**Supplemental Figure 2: Scatterplot of Observed and Predicted ProCNP Concentrations**

**
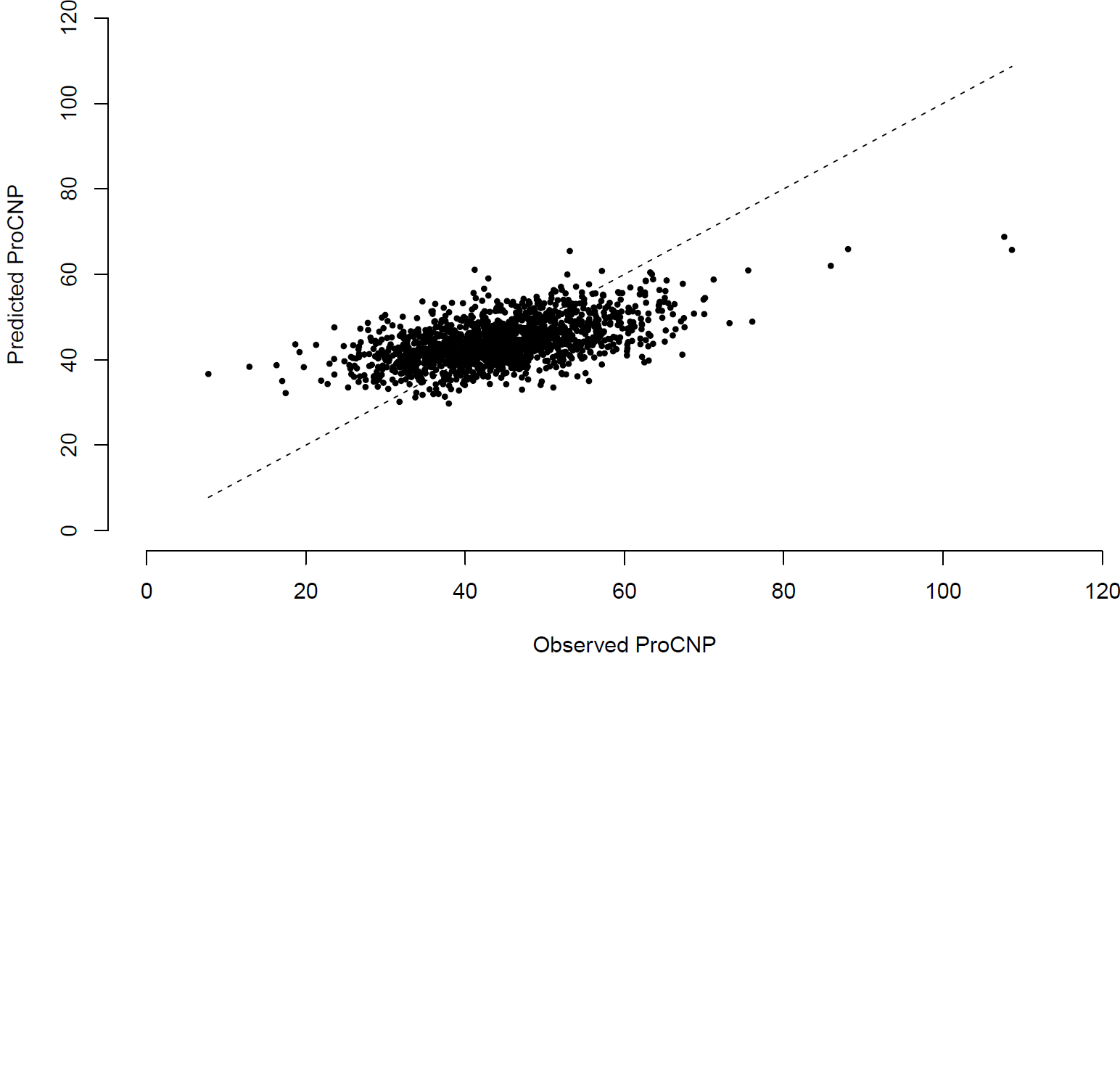
**

The dashed line indicates perfect positive correlation. Concentrations are given in pmol/L.

RECIEVER-OPERATING CHARACTERISTIC CURVE

In the receiver-operating characteristic curve (Supplemental Figure 3) examining the discriminative value of proCNP on the occurrence of death of any cause, the area under the curve was 0.51. As expected, the crude discriminative is poor, where many other factors not captured by proCNP measurement affect the occurrence of death of any cause.

**Supplemental Figure 3: Receiver-Operating Characteristic Curve Displaying Specificity and Sensitivity of ProCNP Concentration in Plasma to Predict All-cause Death.**


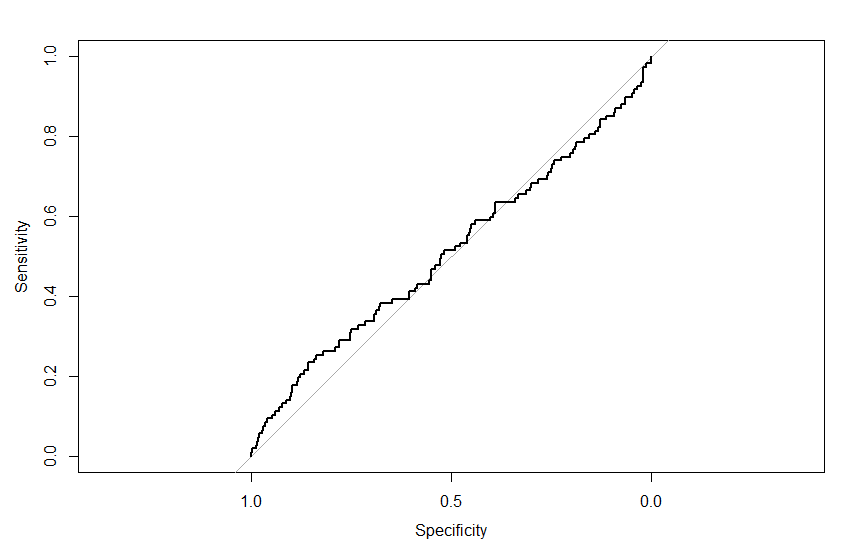

Supplement: Supplemental Material [file mmc1.docx]
